# Editorial

**Published:** 1868-12

**Authors:** 


### Editorial.

APOLOGY.—The *St. Louis Medical and Surgical Journal* calls our attention to the fact, that the October number of the EXAMINER contained among its *selected* articles three that were copied from that Journal without the usual credit. Having a good proof-reader in the office, we do not require the proof-sheets of selected articles to be submitted to us for examination before they are worked off. The articles copied from the *St. Louis Journal* were marked and given to the printer with the expectation that they would be credited according to the standing rule of the office. And we had not noticed the omission until our attention was called to the matter, by a rather sharp rebuke in the latter Journal.

TO SUBSCRIBERS.—This number being the last of the volume, we have directed bills to be sent to all subscribers who are in arrears, stating the amount due up to January 1st, 1869. If any mistakes have been made, we shall be happy to correct them.

In the future, as heretofore, we shall endeavor to make the EXAMINER worthy of the patronage of the profession.

---

CHICAGO MEDICAL COLLEGE.—The friends of a more methodical and extended system of medical education will be glad to learn that the adoption of a full six months' Lecture term, a complete gradation of Classes, and three courses of Lectures before graduation, by this College, has not prevented it from receiving the patronage of an excellent class of students during the present term. The present arrangement proves highly satisfactory to both Faculty and students.

---

DETROIT MEDICAL COLLEGE.—We see, by the Announcement of this new medical school, that its first preliminary course of Lectures commenced on the 3d of November; and the first *regular* course is to commence on the 2d of February next, and continue four months.

Every enlightened physician will concede that Detroit is the only proper place for a medical college in the State of Michigan, simply because it is the only one in which adequate facilities for clinical instruction can be found. But we think our friends engaged in the enterprise there, have committed a great mistake in clinging to the old system of pretending to teach the whole field of medicine in *four months*, and disregarding both classification of students and preliminary education. By this course they present no claims on the patronage of the profession superior to the most ordinary schools in the country; and, with the Medical Department of the State University at Ann Arbor on one side, and the Buffalo Medical College on the other, their chances of success are not brilliant. Had they, at the outset, adopted a complete college organization in accordance with the real interests and often expressed wishes of the profession generally, by making the regular lecture term six months; dividing the branches into series corresponding with the number of years required for study; exacting thorough examinations at the close of each series; with a moderate standard of preliminary education, they would have presented a strong claim on the active sympathy and support of the profession at home and abroad. With this, and the advantage of clinical facilities, they could have competed successfully with their principal rival at Ann Arbor, and ultimately compelled a union, by the removal of the Medical Department of the University to Detroit.

We do not make these remarks from any feeling of unfriendliness towards the Faculty of the Detroit Medical College, for we wish them abundant success in any enterprise that is calculated to promote the educational interests of our profession.

---

Aitken's Science and Practice of Medicine.—We have just received the second volume of the second American edition of this work.

With the many valuable additions made by the editor, Dr. Clymer, this edition presents, perhaps, the most complete and extensive work on practical medicine now before the profession in our language. The publishers, Lindsay & Blakiston, of Philadelphia, have done their part of the work in excellent style.

For sale by S. C. Griggs & Co., Chicago. Price, $12.00, complete.

---

Mortality for the Month of October, 1868:—

The Superintendent's monthly report showed a total of 445 deaths, during October, 29 of which were from convulsions, 17 from cholera infantum, 21 from scarlet fever, 14 from dysentery, 12 from diarrhœa, 9 from diptheria, 12 from croup, 34 from typhoid fever, 35 from phthisis pulmonalis, 16 from pneumonia, 10 from whooping cough, 14 from tabes mesenterica, 14 from teething, 10 from old age, and 6 from causes unknown. There were 20 premature births, and 50 still-births.

COMPARISON.

Deaths in Oct., 1868, ... 445 | Deaths in Oct., 1867, ... 428 | Increase, ... 17

Deaths in Sept., 1868, ... 739 | Decrease, ... 294

AGES.

| | | | | | |
|---|---|---|---|---|---|
| Under 1 | 116 | 20 to 30 | 47 | 70 to 80 | 16 |
| 1 to 3 | 77 | 30 to 40 | 37 | 80 to 90 | 5 |
| 3 to 5 | 35 | 40 to 50 | 30 | Unknown | 1 |
| 5 to 10 | 32 | 50 to 60 | 11 | | |
| 10 to 20 | 23 | 60 to 70 | 15 | Total | 445 |

| | | | | | |
|---|---|---|---|---|---|
| Males, | 260 | Females, | 185 | Total, | 445 |
| Single, | 331 | Married, | 114 | Total, | 445 |
| White, | 443 | Colored, | 2 | Total, | 445 |

NATIVITY.

| | | | | | |
|---|---|---|---|---|---|
| Foreign Chicago | 118 | England | 8 | Sweden | 16 |
| Chicago | 74 | Germany | 68 | Switzerland | 3 |
| Other parts U. S. | 75 | Holland | 2 | Unknown | 4 |
| Austria, | 1 | Ireland | 51 | | |
| Bohemia | 7 | Norway | 5 | Total, | 445 |
| Canada | 6 | Scotland | 4 | | |
| France | 2 | Italy, | 1 | | |

MORTALITY BY WARDS FOR THE MONTH.

| Ward. | Mortality. | Pop. in 1868. | One death in | Ward. | Mortality. | Pop. in 1868. | One death in |
|---|---|---|---|---|---|---|---|
| 1 | 11 | 11,991 | 1,091 | 14 | 40 | 14,168 | 354 1-5 |
| 2 | 15 | 13,739 | 916 | 15 | 37 | 20,429 | 552 1-7 |
| 3 | 15 | 16,620 | 1,108 | 16 | 16 | 16,011 | 1,007 |
| 4 | 18 | 16,499 | 916 3-7 | Armory, | 1 | | |
| 5 | 24 | 13,434 | 559 7-8 | Bridewell, | 2 | | |
| 6 | 30 | 12,507 | 416 8-9 | County Jail, | 1 | | |
| 7 | 62 | 21,957 | 353 1-3 | County hosp. | 13 | | |
| 8 | 29 | 14,003 | 482 5-6 | Chi. River, | 3 | | |
| 9 | 20 | 18,050 | 902 3-5 | Mercy Hosp. | 1 | | |
| 10 | 12 | 13,644 | 1,137 | Marine Hosp. | 1 | St. Mary,s Hosp. | 1 |
| 11 | 25 | 13,317 | 532 3-5 | Soldier's Ho. | 1 | Hosp. for Women and Children, | 1 |
| 12 | 27 | 14,739 | 549 3-5 | Immigrants | 15 | | |
| 13 | 20 | 11,113 | 555 3-5 | | | Home forFriendless, | 4 |

Total, ... 445

The health of the City during the month of October has been very good, comparing favorably with the corresponding month in 1867, when the increase of population is borne in mind. There is a marked diminuition in the infantile mortality, with a decided increase of deaths by scarlet and typhoid fevers. In no other respect is there anything unusual. The influence of the want of drainage is also marked.

The report was adopted and the Board then adjourned.

A Blow to the Fungus Theory of Disease.—In a short communication to the *Centralblatt*, Drs. Bergmann and Schmiedeberg describe a crystalline substance, to which they have applied the name "sulphate of sepine," obtained from putrefying materials, and which they believe represents the proper poison of organic substances undergoing this kind of fermentation. It is obtained by diffusion through parchment-paper, precipitation with corrosive sublimate from an alkaline solution, removal of the mercury of silver, by sulphuretted hydrogen, evaporation and purification of the residue. Large, well-defined, acicular needles are thus obtained, which are deliquescent in the air, and, exposed to heat, melt and carbonize. They possess a powerfully poisonous action. A solution containing scarcely more than one-hundredth of a gramme was injected into the veins of two dogs. Vomiting was immediately induced, and after a short time diarrhœa, which in the course of an hour became bloody. After nine hours the animals were killed, and, on examination, their stomachs and large intestines were found ecchymosed, and the small intestine congested. Frogs could be killed in the same manner.—*Lancet.*—*N. Y. Med. Gazette.*

---

Length of the Colon in Young Children.—At a stated meeting of the New York Obstetrical Society, a specimen of hemicephalus, or anencephalus, was presented by Dr. Jacobi. The child weighed nine pounds. The viscera were well developed, and the colon was unusually long in this case. Dr. Smith made the remark, that he had measured the colon in thirty cases of children under six months, and discovered that from one-quarter to one-third of the large intestine lies below the brim of the pelvis. Dr. Jacobi stated that the descending portion of the colon, in the young infant, was nearly twice the length of that of the adult. It crosses over diagonally towards the right side, instead of lying parallel to the long axis of the body. There is no proper sigmoid flexure as in the adult, but, on account of the great length of the colon, a number of flexures are found.—*Amer. Jour. of Obstetrics.*—*Boston Med. and Surg. Journal.*

---

Pyrethrum for Scabies.—The *Pacific Med. and Surg. Journal* says:—"Flea powder" is composed of the flowers and leaves of *Pyrethrum Roseum*, and probably of *P. Carnosum*, very finely powdered. It is not commonly fatal to insects, but appears to annoy and stupify them. If the article be recent and properly prepared, it is decidedly efficacious. When scattered in the track of ants, it is said to drive them away with certainty. A druggist of this city informs us that he keeps his store almost entirely free from flies by dusting it occasionally on the walls and windows, in places where they are accustomed to alight. The insects disappear when they get the powder in contact with their bodies. It is said that when burnt in small quantities, in a chamber affected with mosquitoes, it destroys them or drives them away. A tincture of the plant, made with dilute alcohol, is recommended as a cure for scabies. The application is said to give prompt relief from itching.—*Med. and Surg. Reporter.*

---

PROFESSORS PANCOAST AND GROSS, of Philadelphia, having lately returned from a tour abroad, the occasion was considered a favorable one to show honor to these distinguished men, and accordingly a public reception took place at the Academy of Music, in that city, on Saturday evening, the 24th ult. The greeting of welcome was delivered by Dr. A. Hewson, and was responded to by the two Professors—the addresses of each being eloquent and appropriate. Addresses were also made by Governor Pollock, Daniel Dougherty, Esq., Dr. Sayre of New York, Dr. Doyle, and others. The whole entertainment is represented as excellently arranged and harmoniously carried out, and reflects credit upon all the parties concerned.—*Boston Medical and Surgical Journal.*

---

STATISTICS OF SPAIN.—The distinguished statistician, Don Ramon de la Sagra, furnishes the following statistics of Spain during the year 1866:—Total population, 15,800,000. Rate of births, 1 to 26; proportion of sexes, 51·65 boys to 48·35 girls. In every 19 births, one was illegitimate; proportion of marriages, 1 to 112 inhabitants. The average number of children to each marriage, as near as can be estimated, is 4·6. Deaths were 1 to 34 of the whole population, 1 to 28 in the cities; 503 of the deaths out of 1000 were under 6 years of age. —*Medical and Surgical Reporter.*

---

A FACT of some interest, if it be confirmed by further experiments, has recently been announced by the French medical journals. M. Telcphe Desmartis, a medical experimenter of Bordeaux, has succeeded, it is said, in inoculating upon plants tubercular matter taken from the human lung. The result has been the production of a particular kind of mycelium. This leads M. Desmartis to establish a comparison between tubercle and sphacelia, or ergot of rye.—*Lancet.—N. Y. Med. Gazette.*

The Structure of the Tactile Corpuscles Demonstrated.—M. Rougct (*London Med. Mirror*) believes that he has succeeded in demonstrating the structure of the tactile corpuscles; from frequent observations he is warranted in saying that the nerve-fibres actually enter the substance of the cone-like corpuscle instead of being merely coiled round it, as was formerly believed. His specimens are prepared by soaking them for some time in acidulated water, and then acting on them with strong nitric acid, which stains the nerve-fibres, but not the other tissues.—*Medical Record.*

---

The Qualifications of an Edinburgh Surgeon in 1505. —In 1505, the surgeons of Edinburgh were in noway behind the other schools of the three kingdoms, and we are somewhat surprised to read that "when the surgeons of Edinburgh were, in 1505, incorporated under the denomination of surgeons and barbers, it was required of them to be able to read and write, to know anatomie, nature, and complexion of every parte of the human bodie, and lykeways to know all vayns of the sayme, that he may make *flewbothomie* in due time together with a *perfect knowledge of shaving beards.*"—*Medical Record.*

---

Mortality of the Abyssinian Expedition.—In the Abyssinian expedition, says the *Deutsche Klinik*, the English troops had 5–8 per cent. of their number sick, and 1–3 per cent. of deaths.

The death-rate among officers was 11 per cent. of the *sick;* among the privates, 18 per cent. in the highlands, and 20 per cent. on the coast and on board the hospital ships. Three medical officers died, and three officers died from violence, one of them a suicide.—*Med. and Surg. Reporter.*

---

Hemorrhage after Extraction of Teeth.—A correspondent of the *Lancet* writes:—"Troublesome hemorrhage sometimes will follow the extraction of a tooth. A case of this kind occurred a short time ago, in which bleeding continued for six or seven hours, until it was stopped by the following treatment, the effect of which is immediate and permanent, and gives no pain. I have treated five cases in the same manner:—Soften a bit of white wax, and mould it into a conical shape, about an inch long, and press it into the cavity, at first lightly, and then very firmly, so as to fill it. Cover this with a thick pad of lint, to retain it in its place, and bind the jaws together for a few hours with some kind of bandage."—*Med. and Surg. Reporter.*

Prof. Peaslee has resigned the chair of Anatomy and Physiology in the Medical Department of Dartmouth College, occupied by him the past 28 years; and has been transferred to the chair of the Diseases of Women. Dr. Lyman B. How, of Manchester, N. H., has been elected to the vacant chair of Anatomy and Physiology.—*Medical Record.*

---

Turpentine as an Antidote to Phosphorus.—The *Archives Gen. de Medecine* calls attention to the custom of the workmen in a match factory at Stafford, who apply phosphorus to the matches, of carrying on their breast a tin cup, containing essence of turpentine. This precaution is said to be sufficient to prevent any ill effects from the action of the phosphorus. It was previously known that the vapor of turpentine prevents the ignition and even the phosphorescence of phosphorus, but the practical application of this knoweledge is not so generally adopted as it should be.—*Bost. Med. and Surg. Jour.*

---

Operation for Hernia without opening the Sac.—In the *New York Medical Record*, Dr. Erskine Mason reports five cases of strangulated hernia, three femoral and two inguinal, which he operated on successfully without opening the peritoneal sac. The method is an old one, but now very generally employed in Great Britain, though not much resorted to in America. Statistics show it to be safer than cutting the sac, which is liable to produce peritoneal inflammation. Dr. Mason thinks it should always be attempted at first, as the sac may be opened afterwards if the hernia cannot be reduced without so doing.

---

In a late discussion before the British Association for the Advancement of Knowledge, several physicians warmly advocated the opinion that the faculty of articulate language resides in the third frontal convolution of the left side of the brain.

---

Money Receipts to Nov. 8th.—Drs. Thos. Whitten, $3; J. C. Allaben, 3; L. B. Cowles, 2; David Prince, 5; J. H. Judson, 3; G. W. Morrill 5; J. A. Adrain, 3; J. H. Hollister, 3; Thos. Hamill, 3; H. N. Hurlbut, 3;

Money Receipts from November 7th to 25th.—Drs. A. H. Salisbury, $3; T. T. Ellis, 3; J. B. Eversole, 3; A. B. Hanna, 3; Hill & Carr, 3; D. R. Taylor, 3; David McDill, 3.

